# Pre-existing astrocytes form functional perisynaptic processes on neurons generated in the adult hippocampus

**DOI:** 10.1007/s00429-014-0768-y

**Published:** 2014-04-19

**Authors:** Marine Krzisch, Silvio G. Temprana, Lucas A. Mongiat, Jan Armida, Valentin Schmutz, Mari A. Virtanen, Jacqueline Kocher-Braissant, Rudolf Kraftsik, Laszlo Vutskits, Karl-Klaus Conzelmann, Matteo Bergami, Fred H. Gage, Alejandro F. Schinder, Nicolas Toni

**Affiliations:** 1 Department of Fundamental Neurosciences, University of Lausanne, 9 rue du Bugnon, 1005 Lausanne, Switzerland; 2 Laboratory of Neuronal Plasticity, Leloir Institute (IIBBA-CONICET), Buenos Aires, Argentina; 3 Department of Fundamental Neurosciences, University of Geneva, Geneva, Switzerland; 4 Department of Anesthesiology, Pharmacology and Intensive Care, University Hospital of Geneva, Geneva, Switzerland; 5 Max von Pettenkofer Institute and Gene Center, Ludwig-Maximilians University Munich, Munich, Germany; 6 Cologne Excellence Cluster on Cellular Stress Responses in Aging-Associated Diseases (CECAD) and University Hospital of Cologne, Cologne, Germany; 7 Laboratory of Genetics, Salk Institute for Biological Studies, La Jolla, CA USA

**Keywords:** Adult neurogenesis, Dentate gyrus, Synaptogenesis, Astrocytes, Perisynaptic processes

## Abstract

The adult dentate gyrus produces new neurons that morphologically and functionally integrate into the hippocampal network. In the adult brain, most excitatory synapses are ensheathed by astrocytic perisynaptic processes that regulate synaptic structure and function. However, these processes are formed during embryonic or early postnatal development and it is unknown whether astrocytes can also ensheathe synapses of neurons born during adulthood and, if so, whether they play a role in their synaptic transmission. Here, we used a combination of serial-section immuno-electron microscopy, confocal microscopy, and electrophysiology to examine the formation of perisynaptic processes on adult-born neurons. We found that the afferent and efferent synapses of newborn neurons are ensheathed by astrocytic processes, irrespective of the age of the neurons or the size of their synapses. The quantification of gliogenesis and the distribution of astrocytic processes on synapses formed by adult-born neurons suggest that the majority of these processes are recruited from pre-existing astrocytes. Furthermore, the inhibition of astrocytic glutamate re-uptake significantly reduced postsynaptic currents and increased paired-pulse facilitation in adult-born neurons, suggesting that perisynaptic processes modulate synaptic transmission on these cells. Finally, some processes were found intercalated between newly formed dendritic spines and potential presynaptic partners, suggesting that they may also play a structural role in the connectivity of new spines. Together, these results indicate that pre-existing astrocytes remodel their processes to ensheathe synapses of adult-born neurons and participate to the functional and structural integration of these cells into the hippocampal network.

**Electronic supplementary material:**

The online version of this article (doi:10.1007/s00429-014-0768-y) contains supplementary material, which is available to authorized users.

## Introduction

Adult hippocampal neurogenesis results in the continuous generation of granule neurons (Altman 1962; Eriksson et al. 1998), which functionally integrate into the hippocampal network. Interestingly, these immature neurons show enhanced excitability and plasticity compared to pre-existing ones (Marin-Burgin et al. 2012; Schmidt-Hieber et al. 2004; Ge et al. 2007; Tronel et al. 2010) and their unique contribution to information processing highlights the role of neurogenesis in hippocampal-dependent learning performances (Kempermann et al. 1997b; Clelland et al. 2009; Sahay et al. 2011; van Praag et al. 1999). More recently, the optogenetic modulation (stimulation or inhibition) of newborn neurons activity in living mice enabled corresponding changes in memory performances (Gu et al. 2012; Alonso et al. 2012), suggesting a critical role for adult-born neurons in complex behavioral tasks. The synaptic integration of adult-born neurons is also of crucial importance for their survival (Toni and Sultan 2011; Bergami and Berninger 2012). Indeed, a loss of *N*-methyl-d-aspartate (NMDA) glutamate receptors reduces the survival of newborn neurons during the period of intense synaptogenesis (Tashiro et al. 2006) and inversely, the stimulation of perforant path fibers enhances the survival of these cells (Stone et al. 2011; Bruel-Jungerman et al. 2006). Therefore, the mechanisms of synaptic integration of adult-born neurons play a critical role in the regulation of adult neurogenesis. We recently showed that adult-born neurons form mature and functional synapses (van Praag et al. 2002; Toni et al. 2007, 2008). However, it is unknown whether synapses formed by new neurons are also ensheathed by astrocytic perisynaptic processes and whether these processes play a role in the function of these newly-formed synapses.

During embryonic development, astrocytes play a crucial role in synapse formation and maturation: synaptogenesis occurs concomitantly with the formation of astrocytes (Catalani et al. 2002; Nixdorf-Bergweiler et al. 1994) and these cells express membrane-bound and also soluble factors that enable synaptic maturation and stability (Pfrieger and Barres 1997; Ullian et al. 2001; Allen et al. 2012; Christopherson et al. 2005). Furthermore, astrocytes extend processes that ensheathe synapses and reuptake glutamate from the synaptic cleft, which is critical not only for synaptic function and plasticity but also for neuronal survival (Rothstein et al. 1996; Tanaka et al. 1997; Volterra and Meldolesi 2005; Faissner et al. 2010). Thus, by providing a molecular and structural scaffold and by regulating glutamate reuptake, astrocytic perisynaptic processes play a crucial role in synaptogenesis that is especially relevant to adult-born neurons. However, the role of astrocytes in synaptogenesis occurring in the adult brain is less clear (Witcher et al. 2007). Shortly after birth, gliogenesis is reduced (Ge et al. 2012) and, although astrocytes remain plastic throughout adulthood (Witcher et al. 2007; Nishida and Okabe 2007; Haber et al. 2006; Hirrlinger et al. 2004), it is unknown whether they interact with synapses formed by adult-born neurons and participate to their function. In particular, the extensive and synchronous synaptogenesis occurring during the maturation of adult-born neurons may not be accompanied by the simultaneous generation of astrocytic processes.

If perisynaptic processes are formed on adult-born neurons, their function and the mechanism of their formation may have crucial implications for the synaptogenesis occurring on these neurons.

## Materials and methods

### Experimental animals

The animals used for microscopy observations were 8- to 10-week-old C57BL6/J, GFAP-GFP or Aldh1l1-GFP mice. C57BL6/J mice were purchased from Janvier (Le Genest Saint Isle, France). GFAP-GFP mice express the green fluorescent protein (GFP) under the control of the astrocyte-specific Glial fibrillary acidic protein (GFAP) promoter (Nolte et al. 2001). These mice were kindly provided by the laboratory of Helmut Kettenmann (Max-Delbruck center for Molecular Medicine, Berlin, Germany). Aldh1l1-GFP mice express GFP under the control of the aldehyde dehydrogenase 1 (ALDH1L1) promoter. These mice were obtained from the GENSAT Project (www.gensat.org) at the Rockefeller University. For electrophysiology experiments, 6- to 7-week-old female C57BL6/J mice were obtained from the Leloir Institute animal facility.

Mice were group-housed in standard cages under light- (12 h light/dark cycle) and temperature-controlled (22 °C) conditions. The maximal number of mice per cage was five. Food and water were available ad libitum. Every effort was made to minimize the number of animals used and their suffering. Experimental protocols were approved by the Swiss animal experimentation authorities (Service de la consommation et des affaires vétérinaires, Epalinges, Switzerland, Authorization number: 2301).

### Virus-mediated labeling

To identify dividing cells and their progenies, we used a retroviral vector derived from the Moloney murine leukemia virus (MoMuLV), containing a red fluorescent protein (RFP)-expression cassette under the control of the cytomegalovirus early enhancer and chicken beta-actin promoter (cag) as previously described (Laplagne et al. 2006; Zhao et al. 2006). For electrophysiological experiments, a cag-GFP retrovirus was used. Final virus titers were 10^7^–10^8^ pfu/mL and 1.5 μL was injected into the dentate gyrus at the following coordinates from the Bregma: anteroposterior −2 mm, lateral 1.75 mm and dorsoventral −2.25 mm. For rabies virus (RABV)-based experiments, two retroviruses (MoMuLV) were used to transduce newly generated neurons in the dentate gyrus: a polycistronic retroviral construct (CAG-*DsRedExpress2*-2A-*G*-IRES2-*TVA)* encoding the transgenes *DsRedExpress2,* the chicken *TVA* receptor and the RABV *glycoprotein* (*G*), and a control virus encoding *DsRedExpress2 and TVA* but lacking *G*, thereby preventing trans-synaptic transfer. Five weeks after retroviral injection, 0.5 μl of RABV was delivered to the dentate gyrus and animals were analyzed 1 week later. The RABV SADΔG-eGFP used for this study was obtained as previously described (Finke et al. 2003; Deshpande et al. 2013). After every injection and throughout the experiment, animals were regularly monitored for their physical recovery in agreement with, and under the approval of the European and German guidelines on animal experimentation.

### Bromodeoxyuridine (BrdU) administration and brain slice preparation

Mice were injected intraperitoneally with 100 mg/kg of BrdU (Sigma-Aldrich, Buchs, Switzerland) three times, at 2-hour intervals. Twenty-eight days later, mice were perfused with 4 % paraformaldehyde in phosphate-buffered saline, their brains were cryoprotected and sectioned at a thickness of 40 μm, and one out of six sections selected for analysis. Slices were first incubated in 50 % formamide in 2x saline sodium citrate buffer (2x SSC) at 65 °C for 2 h, rinsed twice in 2x SSC, incubated in 2 M HCl for 30 min at 37 °C, and rinsed in 0.1 M borate buffer pH 8.5 for 10 min. Sections were then rinsed six times in tris buffer saline for a total time of 90 min.

### Immunohistochemistry

RFP signal was amplified using rabbit anti-RFP IgG (600-401-379 Rockland Immunochemicals, Gilbertsville, Pennsylvania, USA; diluted 1:1,000 in phosphate buffer saline) and Hylite 594 goat anti-rabbit IgG secondary antibody (61056-1-H594 Biotrend Chemicals AG, Wangen, Switzerland;1:500). GFP signal was amplified using Chicken anti-GFP IgG (GFP-1020 Aves Labs, Tigard, Oregon, USA; 1:1000) and Dylight 488 goat anti-chicken IgY (103-485-155 Jackson ImmunoResearch Europe ltd., Suffolk, United Kingdom; 1:500). Lucifer yellow was detected using rabbit anti-Lucifer Yellow IgG (A-5750 Invitrogen, Carlsbad, CA; 1:4000 dilution) and Hylite 594 goat anti-rabbit IgG secondaryantibody. GFAP was detected using rabbit anti-GFAP IgG (180063 Life Technologies Europe B.V., Zug, Switzerland; 1:1000) and goat Alexa fluor 555 anti-rabbit IgG (A21428 Life Technologies Europe B.V., Zug, Switzerland; 1/250) or donkey Alexa fluor 647 anti-rabbit IgG (A31573 Life Technologies Europe B.V., Zug, Switzerland; 1:250). Doublecortin was detected using goat anti-doublecortin IgG (sc-8066 Santa Cruz Biotechnology, Inc., Dallas, Texas; 1:1000) and Alexa fluor 555 donkey anti-goat IgG (A21432 Life Technologies Europe B.V., Zug, Switzerland; 1:250). NeuN was detected using mouse anti-NeuN IgG (MAB377 Chemicon International, Inc., Temecula, California; 1:1000) and Alexa fluor 488 goat anti-mouse IgG (A11029 Life Technologies Europe B.V., Zug, Switzerland; 1:250). BrdU was revealed using rat anti-BrdU IgG (ab6326 Abcam, Cambridge, United Kingdom; 1:250) and goat Alexa fluor 594 anti-rat IgG (A-11007 Life Technologies Europe B.V., Zug, Switzerland; 1:250). S100β was detected using rabbit anti-S100β IgG (ab868 Abcam; 1:500) and donkey Alexa fluor 647 anti-rabbit IgG (A31573 Life Technologies Europe B.V., Zug, Switzerland; 1:250). 4,6 Diamidino-2-phenylindole (DAPI) was used to reveal nuclei.

### Confocal microscopy and image analyses

Hippocampal sections were imaged using a Zeiss LSM 710 confocal microscope (Carl Zeiss, Feldbach, Switzerland). The alignment of the filters and of the detector was controlled with the use of double-labeled fluorescent beads (TetraSpeck Fluorescent Microsphere Standards, diameter: 0.5 µm, Molecular Probes, Zug, Switzerland). Global views of neurons and GFP-labeled astrocytes were imaged with a 40x oil lens and a z-step of 2 µm, and dendrites or mossy fiber terminals were imaged with a 63x oil lens and a z-step of 0.38 µm. Deconvolution was performed with Huygens Essentials for Win64 software (Scientific Volume Imaging B.V, Hilversum, Nertherlands) on dendrite and mossy fiber terminal images. Image acquisition was performed according to Nyquist rate sampling. Voxel size was 0.04 µm × 0.04 µm × 0.38 µm (*X*; *Y*; *Z*). Point spread function was measured in the green and red channels using double-labeled fluorescent beads (diameter 0.5 µm). The quality threshold was 0.1, the signal to noise ratio was 30 and the maximum number of iterations was 40. All analyses were performed using Fiji software (freely available at http://fiji.sc/).

To evaluate the density of astrocytes and doublecortin-positive (DCX-positive) cells, 9 hippocampal slices of Aldh1l1-GFP mice were analyzed on maximal intensity projections of confocal z-stacks imaged with a 40x oil lens and a z-step of 2 µm. Regions of interest including the molecular layer and the granule cell layer of the dentate gyrus were selected based on the apparent density of DCX-positive cells. The density of cells expressing DCX, GFAP or Aldh1l1 was then measured manually in each region.

To determine the number of astrocytes potentially contacting a newborn neuron (Fig. 3), we did not use GFAP immunostaining, which labels only the largest processes. We, therefore, used GFAP-GFP mice to measure the mean radius of astrocytes, i.e., the average of the distance between the nucleus and the border of its territory. We analyzed 37 astrocytes from 6 GFAP-GFP mice. We then used Aldh1l1-GFP mice to assess the number of astrocytic territories crossed by an individual newborn neuron. When the distance between the nucleus of a labeled astrocyte and a dendrite of an adult-born neuron was less than or equal to the average radius of an astrocytic territory, astrocytes were likely contacting the identified neuron.

The maximal dendritic extension was defined as the ratio between the length from the cell body of the neuron to the tip of its longest dendrite and the distance from the cell body to the end of the molecular layer of the dentate gyrus (Supplementary Fig. 1; Krzisch et al. 2013).

Contacts between astrocytic processes, dendritic spines, and mossy fiber terminals were analyzed on z-stacks, after deconvolution was performed. The area and perimeter of mossy fiber terminals (MFT, Supplementary Fig. 3) were measured on maximal intensity projections by tracing the contour of the MFT, excluding the satellites and filopodia. Astrocytic contacts on MFT was calculated as a percentage of the projected MFT area.

### Electron microscopy and analyses

Electron microscopy was performed as previously described (Toni et al. 2008). Briefly, mice were transcardially perfused with 4 % PFA in 0.1 M phosphate buffer, pH 7.4, and maintained at 4 °C overnight. After postfixation for 72 h in the same fixative, 50 µm-thick coronal vibratome sections were cryoprotected and freeze-thawed in liquid nitrogen. After a treatment in 0.3 % hydrogen peroxide (vol/vol, 5 times, 5 min. each) and a block with 0.5 % bovine serum albumin (BSA-C, Aurion), slices were incubated overnight in the primary antibody (rabbit antibody to GFP, 1:500, Chemicon) at 4 °C on a shaker and then incubated for 5 h in biotinylated secondary antibody (goat antibody to rabbit (F)ab fragment, 1:200, Jackson Laboratories). The slices were then incubated for 2 h in avidin biotin peroxidase complex (ABC Elite, Vector Laboratories), followed by 3,3′-diaminobenzidine tetrachloride (DAB, Vector Laboratories Kit) for 10 min. The sections were then postfixed overnight in 2.5 % glutaraldehyde (wt/vol), followed by 4 % osmium tetroxide for 1 h, dehydrated in ascending concentrations of ethanol and then acetone, and embedded in Epoxy resin. Forty to 150 serial sections were cut at 50-nm thickness and collected on single-slot grids and imaged with a digital camera (MegaView III, SIS) mounted on a JEOL 100 CXII transmission electron microscope at a 19,000× magnification.

### Electrophysiological recordings

CAG-GFP expressing retrovirus was stereotactically delivered to the dentate gyrus of 6-week-old female mice. Mice were anesthetized and decapitated at 4–5 weeks post-injection. Brains were removed into a chilled solution containing (mM): 87 NaCl, 25 NaHCO_3_, 25 glucose, 75 sucrose, 2.5 KCl, 1.25 NaH_2_PO_4_, 0.5 CaCl_2_, and 7 MgCl_2_. The right hippocampus was dissected and coronal slices (400 µm thick) were cut with a vibratome (Leica VT1200 S, Nussloch, Germany) and transferred to a chamber containing artificial cerebrospinal fluid (ACSF; mM): 125 NaCl, 2.5 KCl, 2 NaH_2_PO_4_, 25 NaHCO_3_, 2 CaCl_2_, 1.3 MgCl_2_, 1.3 Na^+^-ascorbate, 3.1 Na^+^-pyruvate, and 10 dextrose (315 mOsm) bubbled with 95 % O_2_/5 % CO_2_. Slices were incubated at 30 °C for 45 min and then stored at room temperature until use. Recorded neurons were visually identified by fluorescence and infrared DIC videomicroscopy. Whole-cell recordings were performed using microelectrodes (4–5 MΩ) filled with (mM): 150 k-gluconate, 1 NaCl, 4 MgCl_2_, 0.1 EGTA, 10 HEPES, 4 ATP-tris, 10 phosphocreatine, and 0.3 GTP-tris. All recordings were obtained using Axopatch 200B amplifiers (Molecular Devices, Sunnyvale, CA), digitized (Digidata 1322A, Molecular Devices), and acquired at 20 kHz onto a personal computer using the pClamp 9 software (Molecular Devices). Membrane capacitance and input resistance were obtained from current traces evoked by hyperpolarizing steps (10 mV). Excitatory postsynaptic currents (EPSCs) were recorded in the presence of picrotoxin (100 μM) and final CaCl_2_ 3 mM at a holding potential of −70 mV in response to low frequency (0.07 Hz) stimuli delivered to the perforant path. Dihydrokainate (DHK, 300 µM) was bath-applied after a stable peak-amplitude baseline was recorded. Criteria to include cells in the analysis were visual confirmation of GFP in the pipette tip, absolute leak current <50 pA at −70 mV, and reversibility of the effect after drug washout. Series resistance was typically 20–25 MΩ, and experiments were discarded if >40 MΩ.

### Statistical analyses

Hypothesis testing was two-tailed. All analyses were performed using GraphPad Prism 6 (Graphpad Software, Inc., La Jolla, California, USA). First, Shapiro–Wilk tests were performed on each group of data to test for distribution normality. When the distribution was not normal, non-parametric Kruskal–Wallis test was applied. Otherwise, the analysis was performed using parametric tests: one-way Analysis of Variance followed by a post hoc unpaired *t* test and Bonferroni correction. For two-sample comparisons, when the distribution was normal, the equality of variances of the groups was tested and the adequate unpaired *t* test was used. When the distribution was not normal, a Mann–Whitney test was applied. Pearson’s correlation test was applied on *XY* scatters when the distribution of data was normal and, if not, Spearman’s correlation test was performed. For the statistical analysis of normalized peak EPCS and normalized paired pulse ratio (PPR), paired comparisons were performed between averaged data points corresponding to the initial (baseline) and final (DHK), 5 min for each experiment. Data are presented as mean ± SEM.

## Results

### Astrocytic perisynaptic processes formation on new neurons

**Fig. 1:**
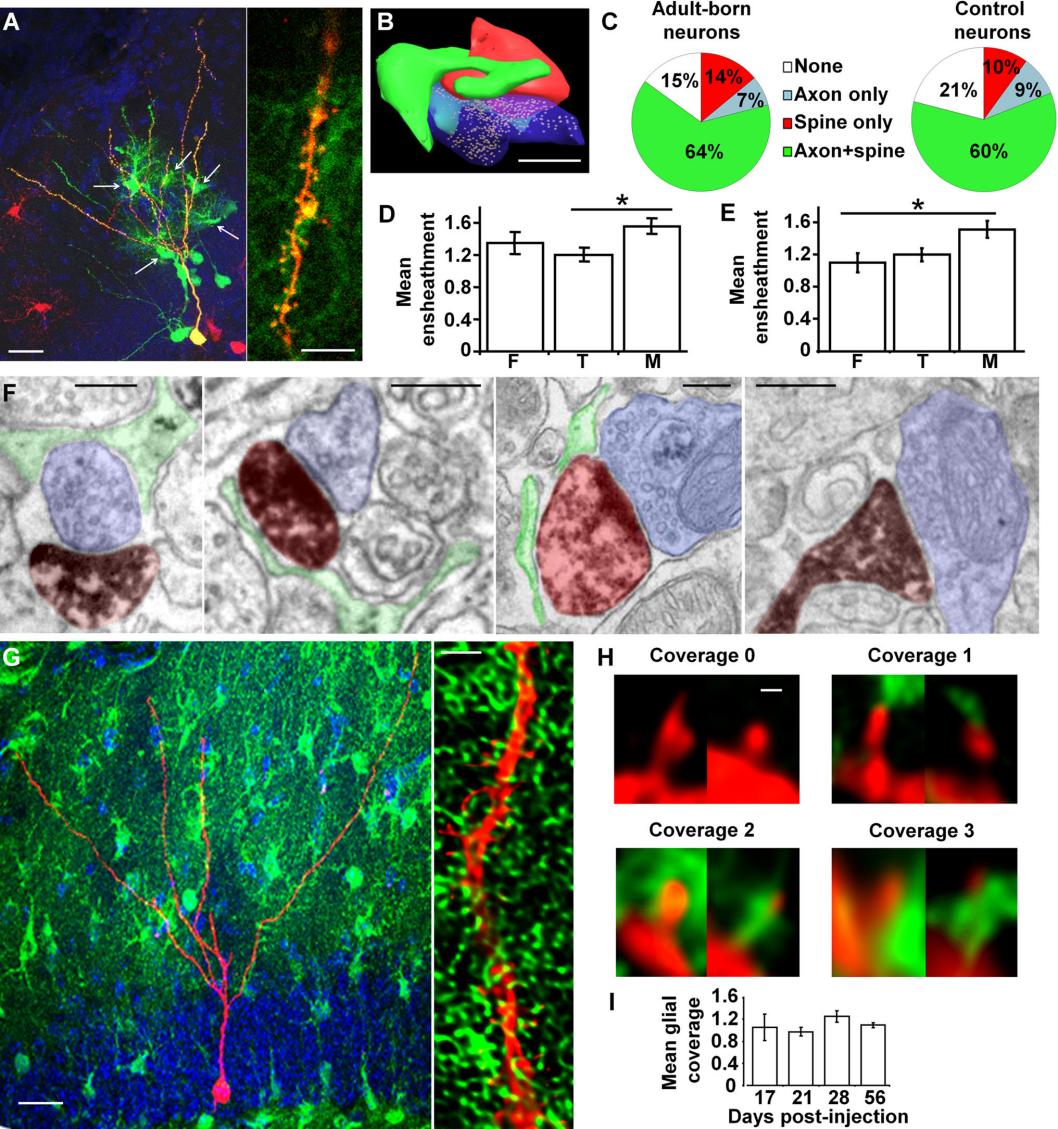
Astrocytic perisynaptic processes ensheathe dendritic spines of adult-born neurons. **a** *Left* RABV used for trans-synaptic tracing reveals astrocytes: Mice were infected with a MoMuLV expressing DsRed, protein G and TVA. Five weeks later, they were infected with RABV expressing GFP and examined 1 week later. Confocal micrograph maximal projection reveals GFP-labeled astrocytes (*arrows*) contacting a starter neuron (*yellow*) co-infected by MoMuLV and RABV. Presumable presynaptic neurons (RABV-only infected) are also labeled in *green*. *Scale bar* 20 µm. *Right* higher magnification single optical section micrograph showing the dendrite of a starter neuron (*yellow*) in close contact with GFP-labeled astrocytic processes. *Scale bar* 5 µm. **b** Three-dimensional reconstruction of a synapse between a dendritic spine (*red*) and an axon terminal (*blue*) ensheathed by an astrocytic perisynaptic process (*green*). *Scale bar* 0.25 µm. **c** Proportion of the different types of astrocytic ensheathement on spines of adult-born neuron synapses (*left*) and GFP-negative, control neurons (*right*). **d** Mean perisynaptic ensheathement on spines of different morphologies on adult-born neurons (Kruskal–Wallis test, *p* < 0.05, *n* = 50–100 spines per group). **e** Mean perisynaptic ensheathement on spines of different morphologies on control neurons (Kruskal–Wallis test, *p* < 0.05, *n* = 50–100 spines per group). *F* filopodia, *T* thin spines, *M* mushroom spines. **f** Electron micrographs of dendritic spines from newborn neurons (false-colored in *red*) and axon terminals (*blue*) ensheathed by an astrocytic perisynaptic process (*green*). From left to right: the perisynaptic process is found on the axon terminal only, on the dendritic spine only, on both synaptic partners or is absent. *Scale bars* 0.25 µm. **g** *Left panel* confocal micrograph maximal projection of an adult-born neuron (*red*) in the dentate gyrus of an Aldh1l1-GFP mouse, 4 weeks post-injection. *Scale bar* 20 µm. *Right panel* confocal micrograph single optical section (after deconvolution) of a dendrite of a newborn neuron (*red*) in the dentate gyrus of an Aldh1l1-GFP mouse 4 weeks post-injection. *Scale bar* 2 µm. **h**. Confocal micrographs of dendritic spines with astrocytic processes scored in four classes: score 0 no coverage, score 1 coverage inferior or equal to 25 % of the spine head, score 2 coverage between 25 and 50 %, score 3 coverage above 50 %. *Scale bar* 0.5 µm. **i** Histogram of the mean perisynaptic ensheathement on neurons of different ages, as assessed by confocal microscopy [One-way ANOVA between groups, *F*(3,71) = 1.18, *p* = 0.32, *n* = 84–1,780 spines per group]

We first assessed whether dendritic spines of adult-born neurons were ensheathed by astrocytic processes. Recently, a rabies virus (RABV)-based approach for mono-synaptic tracing of the inputs on adult-generated neurons unexpectedly resulted in the labeling of astrocytes (Deshpande et al. 2013; Vivar et al. 2012). To examine whether this RABV-mediated labeling of astrocytes resulted from a direct contact with new neurons, we transduced newborn neurons with a DsRed-encoding Moloney Murine Leukemia virus (MoMuLV; G-TVA retrovirus) co-expressing the EnvA receptor TVA (required for RABV internalization) and the RABV glycoprotein (G) required for subsequent synaptic transfer. Control injections were performed with a virus expressing DsRed and TVA, but not G. Five weeks later, mice were injected with an EnvA-pseudotyped RABV encoding for GFP and analyzed 1 week thereafter. On average 1.02 ± 0.21 RABV-labeled astrocytes were found per neuron in G-TVA retrovirally injected mice. This number is underestimated, since RABV-mediated trans-synaptic tracing does not label all presynaptic partners. In contrast, only 0.02 ± 0.02 labeled astrocytes were found in control virus-injected mice (*p* < 0.05, *n* = 3 mice, Student’s *t* test). Moreover, about 90 % of secondarily infected astrocytes had visible dendrites of RABV-infected new neurons crossing their territories (Fig. 1a), suggesting that astrocytes formed direct contacts with the dendrites of adult-born neurons.

We then searched for direct evidence of astrocytic perisynaptic processes on spines from adult-born neurons. Mice were injected with a GFP-encoding retrovirus (MoMuLV) to identify newborn neurons and 1 month later, hippocampal slices were immunostained against GFP and observed using electron microscopy. Astrocytic processes were identified on serial sections, based on their clear cytoplasm, the irregular shape of their fine processes and the presence of glycogen granules in their largest processes. A perisynaptic ensheathment was defined by the apposition between an astrocytic process and a synapse, for a length of at least 100 nm (Fig. 1b–f). Of 100 dendritic spines analyzed (in 4-week-old granule neurons), a perisynaptic ensheathment was present on 85 synapses and the astrocytic process was either apposed to the axon terminal, the dendritic spine, or both (Fig. 1c, f). The presence and position of the perisynaptic processes were similar between spines from newborn neurons and spines from GFP-negative, control neurons (100 spines analyzed, Fig. 1c). Upon maturation, dendritic protrusions transition from filopodia to thin spines and mushroom spines (Toni et al. 2007). To test whether perisynaptic ensheathment varied with spine morphology, we estimated the extent of ensheathement on 3D reconstructions in 4 categories, as follows: 0 (no coverage); 1: 1–25 %; 2: 26–50 %; 3: >50 % of the surface area of the spine head. We did not find any difference between mean glial coverage of synapses on newborn neurons and on GFP-negative neurons (Mann–Whitney test, *p* = 0.10). Larger dendritic spines had a slightly more important astrocytic ensheathment (Fig. 1d, e, Kruskall-Wallis tests, *p* < 0.05), similarly to dendritic spines of the CA1 stratum radiatum (Witcher et al. 2007).

To test whether perisynaptic ensheathment depended on the age of the new neurons, we next used confocal microscopy. Astrocytes were identified with the use of Aldh1l1-GFP mice, which express GFP in about 95 % of astrocytes, a number that is probably underestimated, as it was assessed by immunohistochemistry against GFAP (Supplementary Fig. 2). New neurons were identified with a MoMuLV expressing the red fluorescent protein (mRFP1) and dendritic spines were examined at different time points after viral injection (17, 21, 28, 56 days post-injection (dpi), *n* = 20–36 neurons per time point, Fig. 1g–i). The extent of perisynaptic coverage was estimated in four categories as described above (Fig. 1h). On a total of 2,984 spines, 56 % were in contact with a perisynaptic process. We found no age-related difference in the mean proportion of astrocytic coverage (One-way ANOVA between groups, *F*(3,71) = 1.18, *p* = 0.32, Fig. 1i). Thus, astrocytic perisynaptic processes ensheathe spine synapses of adult-born neurons and the frequency and distribution of these processes are similar to those of GFP-negative, control neurons and independent of spine morphology or neuronal age.

**Fig. 2:**
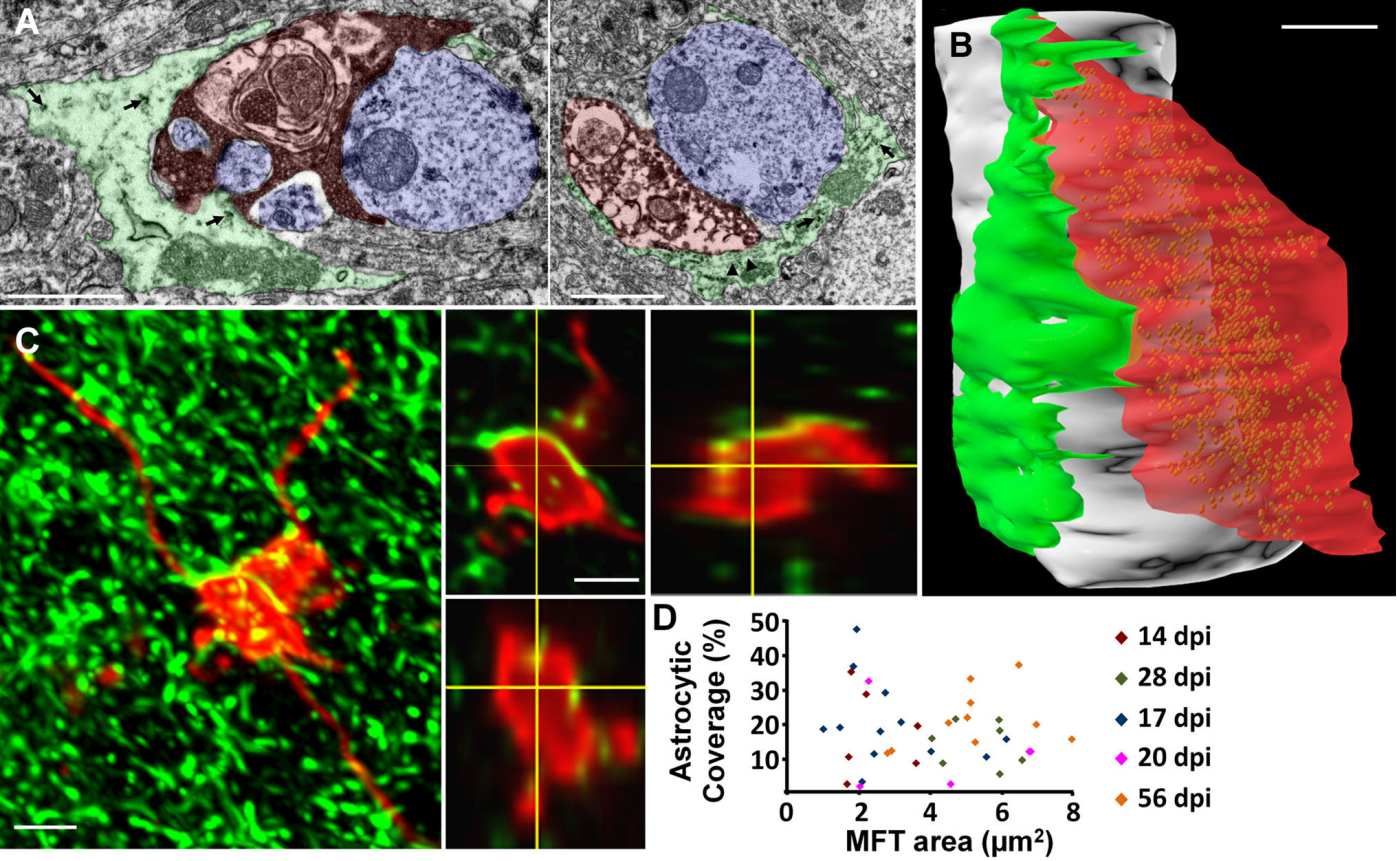
Astrocytic perisynaptic processes ensheathe MFT of adult-born neurons. **a** Electron micrographs of astrocytic processes (false-colored in *green*) ensheathing synapses between axon terminals of 56 dpi adult-born neurons (*red*) and dendrites or thorny excrescences of CA3 pyramidal neurons (*blue*). The perisynaptic ensheathement covers the presynaptic bouton (*left panel*) or both the presynaptic terminal and the dendrite (*right panel*). *Arrows* indicate glycogen granules; arrowheads indicate cytoskeletal filaments. **b** Three-dimensional reconstruction of a synapse between the dendrite of a CA3 pyramidal neuron (*white*) and the axon terminal of a newborn neuron (*red*), ensheathed by an astrocytic process (*green*). **c** Confocal micrographs showing contacts between the MFT of a 56 dpi adult-born neuron and astrocytic processes. *Left panel* maximal intensity projection (after deconvolution). *Right panel* single focal plane and orthogonal projections. **d** Distribution of the proportion of the surface of MFT covered by astrocytic processes, as a function of MFT area and neuronal age (Spearman’s Rank correlation test, *p* = 0.25, *R* ^2^ = 0.01, *n* = 5–12 MFT per timepoint). *Scale bars* in **a** and **b** 1 µm, in **c** 2 µm

Finally, we examined the astrocytic ensheathment on the efferent synapses of the new neurons in the CA3 area, i.e., the mossy fiber terminals (MFT). Using serial-section electron microscopy, we analyzed 27 MFT and found that all but one were ensheathed by astrocytic perisynaptic processes (Fig. 2a, b) that covered either the terminal only (1 of 26 boutons, Fig. 2a left panel) or both the dendrite and the terminal (25 of 26 boutons, Fig. 2a right panel and Fig. 2b). Consistently with previous observations in the CA3, astrocytic processes were tightly apposed to the MFT or the dendritic shaft but never touched thorny excrescences or synaptic clefts, which were engulfed in the MFT (Rollenhagen et al. 2007). Using confocal microscopy, we then examined whether perisynaptic processes changed with neuronal age. We examined 64 MFT of adult-born neurons at 14, 17, 21, 28 and 56 dpi (5–12 MFT per mouse, 1–2 mice per time point, Fig. 2c, d) and measured the percentage of the projected surface area of the MFT covered by astrocytic processes. On average, 24.6 ± 1.6 % of the surface area of terminals was covered by astrocytic processes. Although the projected area of the MFT increased with neuronal age (Supplementary Fig. 3), the proportion of MFT area covered by astrocytic processes remained constant (Spearman’s Rank correlation test, *p* = 0.25, *R* ^2^ = 0.01, Fig. 2d). Thus, perisynaptic processes were present on MFT and their coverage did not depend on MFT size or neuronal age.

Together, these results show that both input and output synapses of adult-born neurons are ensheathed by perisynaptic processes, which are indistinguishable from perisynaptic processes of GFP-negative neurons. Furthermore, the extent of the astrocytic coverage does not vary with synapse size or neuronal age, suggesting an early formation of the perisynaptic processes upon dendritic spine or axon terminal formation.

### Contribution of pre-existing astroglia to the ensheathment of synapses from new neurons

**Fig. 3:**
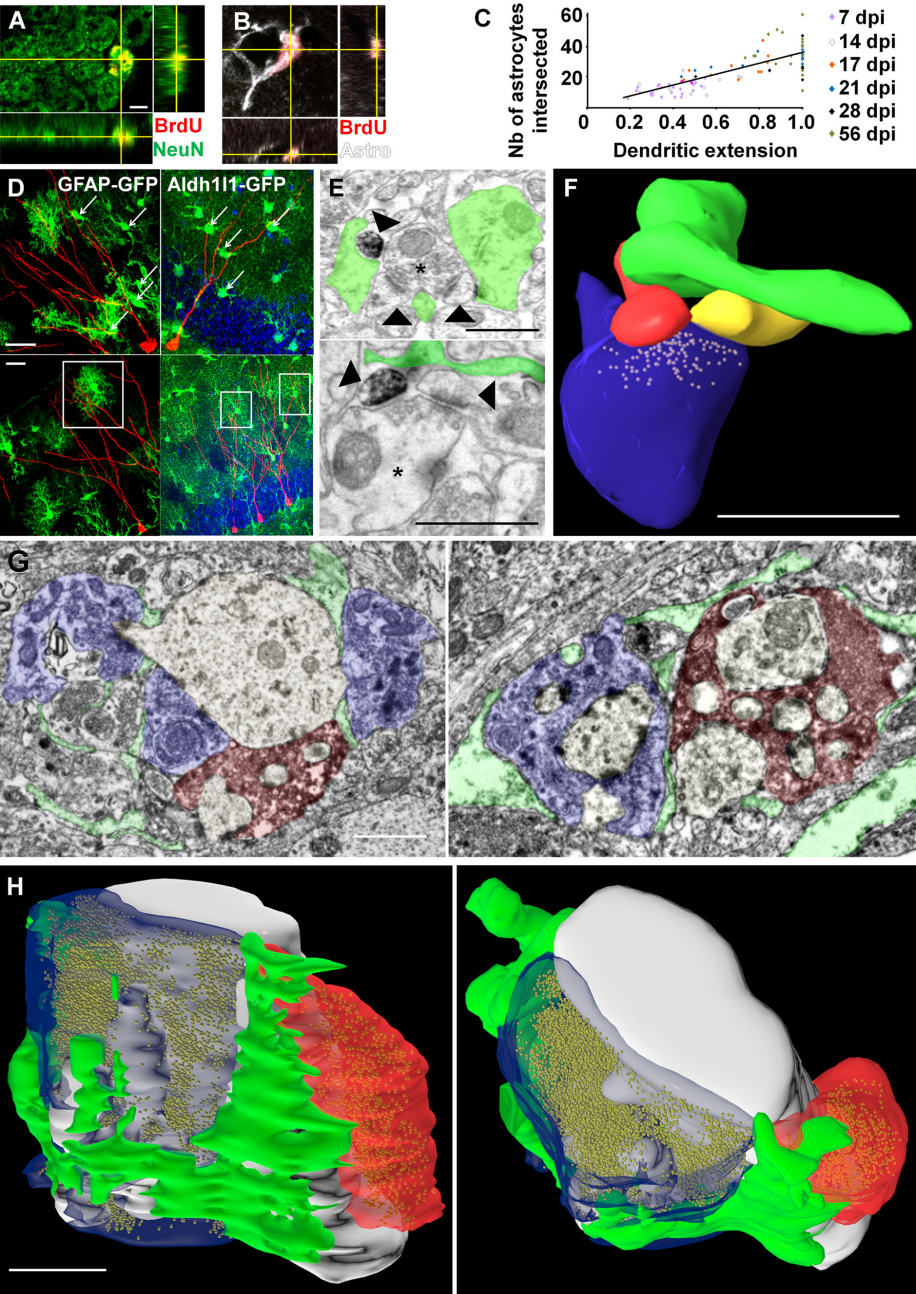
Pre-existing astrocytes ensheathe synapses from adult-born neurons. **a, b** Confocal micrographs and orthogonal projections of hippocampal sections immunostained for BrdU and NeuN (**a**) and BrdU and GFAP/S100β (Astro). (**b**). *Scale bar* 5 µm. **c** Scatter plot showing the number of astrocytic territories intersected by newborn neurons, as a function of their normalized dendritic extension (Spearman’s rank correlation test, *p* < 0.0001, *R* ^2^ = 0.64, *n* = 6–26 neurons per timepoint, 85 neurons in total). **d** Confocal micrographs of newborn neurons (*red*) in GFAP-GFP mice (*left panels*) and Aldh1l1-GFP mice (*right panels*), illustrating one neuron intersecting the territory of several astrocytes (*upper panels, arrows*) and several neurons intersecting the territory of the same astrocyte (in *white squares*, *lower panels*). *Scale bars* 20 µm. **e** Electron micrographs of MSBs (*stars*) formed by the spine of a newborn neuron (dark immunolabeling) and ensheathed by a perisynaptic process (false-colored in *green*). *Arrowheads* point to dendritic spines. **f** 3D reconstruction of the MSB illustrated in the *lower* *panel* of **e** *Scale bars* in **e** and **f** 0.5 µm. **g** Electron micrographs of MFT from new neurons (dark immunolabelling, false-colored in *red*) synapsing with the dendrite or thorny excrescence of a CA3 pyramidal cell (not colorized) that synapse with another non-labeled MFT (*blue*). The perisynaptic processes are colored in *green*. **h** 3D reconstruction of a perisynaptic process (*green*) ensheathing a MFT from a new neuron (*red*) and from a non-labeled neuron (*blue*). *Scale bars* in **g** and **h** 1 µm

Adult neurogenesis also generates new astrocytes and perisynaptic processes on adult-born neurons may originate from astrocytes generated concomitantly or before a given adult-born neuron. To address this question, we first examined whether astrocytes generated concomitantly to adult-born neurons were sufficient to ensheathe all synapses from newly formed neurons, by comparing the number of adult-born astrocytes with the number of astrocytic territories spanned by newborn neurons. Gliogenesis and neurogenesis were evaluated with the injection of the cell proliferation marker BrdU followed, 28 days later, by immunohistochemistry against BrdU, GFAP, S100β and NeuN (Fig. 3a, b). The newly-generated GFAP^+^-S100β^+^ astrocytes to Neu-N^+^ neurons ratio was 0.13 ± 0.03. Although GFAP immunostaining does not label all astrocytes, this ratio is consistent with previous observations (Kempermann et al. 1997a). We then measured the dimensions of individual astrocytes using GFAP-GFP mice and found that on average, astrocytes had a radius of 43 ± 1.5 μm (*n* = 37 astrocytes), as measured from the nucleus to the edge of the territory. We then used Aldh1l1-GFP mice to count the number of astrocytic territories intersected by new neurons, identified by the retroviral approach. The number of intersected territories increased with the age of new neurons and correlated with the extension of their dendritic tree (Spearman’s rank correlation test, *r* ^2^ = 0.64, *p* < 0.0001, Fig. 3c), averaging 20 ± 0.13 astrocytes. Thus, although several new neurons occasionally intersect the same astrocytic territory (Fig. 3d, lower panel), astrocytes generated at a given time during adulthood were not sufficient to ensheath all synapses from neurons generated simultaneously, suggesting that new neurons intersect primarily the territories of pre-existing astrocytes.

To directly assess the possibility that adult-born neurons recruit pre-existing perisynaptic processes, we examined the distribution of perisynaptic processes between new and pre-existing synapses. We recently reported that during their synaptic integration, new neurons preferentially contact pre-existing synapses, thereby forming multiple synapse boutons (MSB). More specifically, at 30 dpi, over 60 % of all spines contact MSB (Toni et al. 2007). At this time point, we analyzed 50 MSB with serial-section electron microscopy and found that 27 (54 %) displayed an astrocytic perisynaptic process that ensheathed both the dendritic spine of the new neuron and the dendritic spine of the pre-existing neuron (Fig. 3e, f). Similarly, in the CA3 area, 20 of 22 (91 %) MFT from new neurons shared an astrocytic process with at least one non-labeled MFT contacting the same dendrite (Fig. 3g, h). Thus, perisynaptic processes were shared between neurons of different ages, suggesting that processes ensheathing pre-existing synapses modify their structure to ensheathe newly-formed synapses.

Together, these results suggest that new neurons intersect principally the territories of pre-existing astrocytes, which remodel their processes to ensheathe the newly-formed synapses.

### Functional role of perisynaptic processes on adult-born neurons

**Fig. 4:**
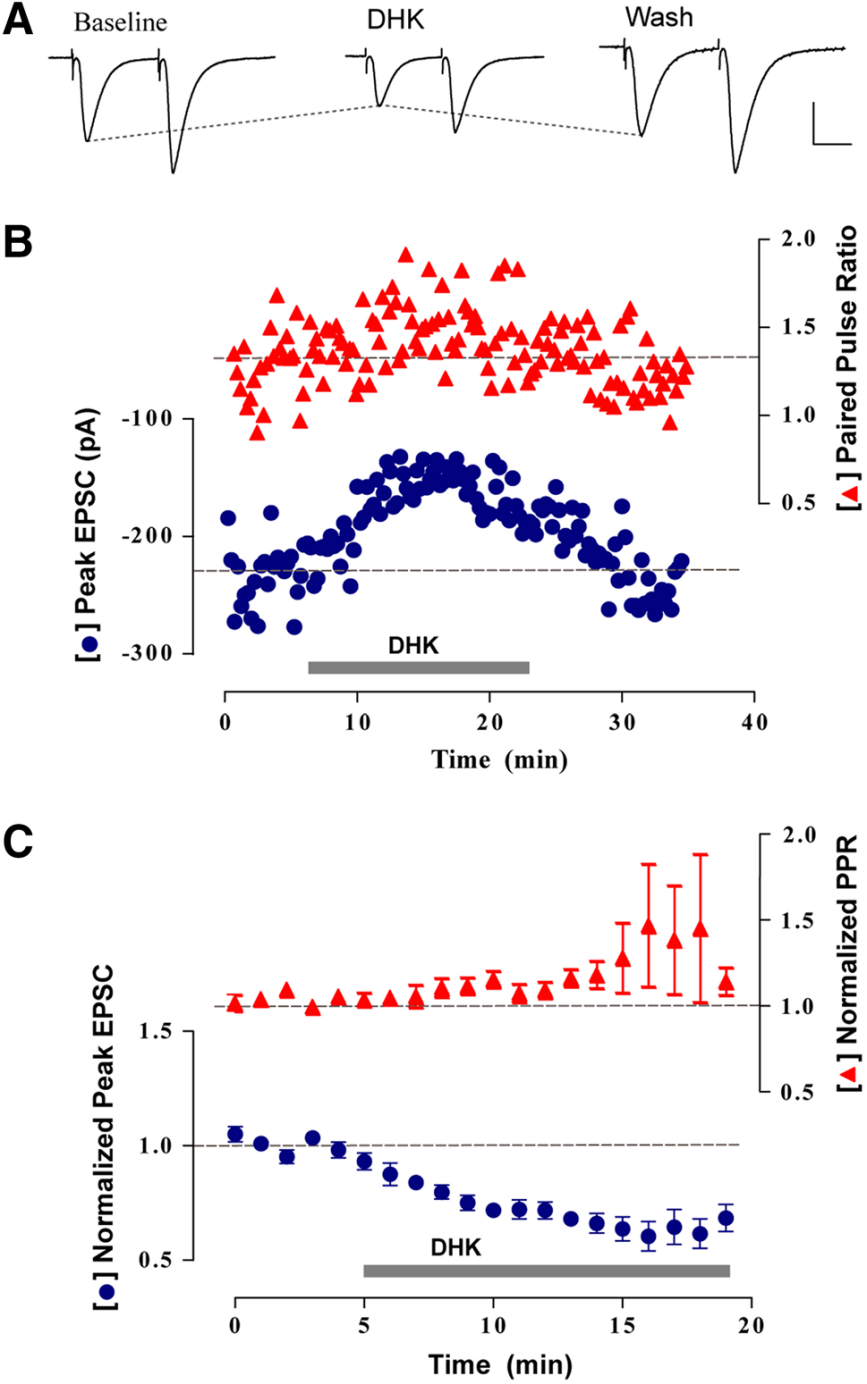
Astrocytic modulation of glutamatergic synaptic transmission on adult-born neurons. **a**, **b** Representative experiment showing whole-cell recordings obtained from a retrovirally labeled 4-week-old neuron. Paired pulses (50 ms apart) were delivered to the perforant path at low frequency (0.07 Hz) in the presence of picrotoxin (100 µM). **a** Postsynaptic current traces obtained before (Baseline, at 4 min), during (DHK, at 17 min) and after (Wash, at 32 min) application of DHK (300 µM). Each trace is an average of 12 sweeps. *Scale* *bars* 100 pA, 20 ms. **b** Peak amplitude of the first EPSC and paired-pulse ratio (PPR) measured in the same experiment shown in **a**. *Dotted lines* denote mean baseline values. **c** Mean values obtained from nine experiments showing normalized peak EPSCs and PPR. Data points were binned in 1-min intervals. *Dots* indicate mean ± SEM values. DHK reduced peak EPSC amplitude (0.64 ± 0.06, *p* < 0.01, Wilcoxon test for paired samples). PPR increase was consistent but not significant (1.33 ± 0.26, *p* = 0.16, Wilcoxon test for paired samples). *Gray bars* denote DHK (300 µM) application

It is conceivable that the observed physical interactions between perisynaptic astrocytic processes and nascent synapses play both structural and functional roles in the synaptic connectivity of newborn neurons. For instance, it is well known that astrocytic glutamate transporters exert a tight control of glutamate clearance at the synapse (Huang and Bergles 2004). Perisynaptic glutamate uptake can shape the strength and kinetics of postsynaptic currents, which in turn determines the responsiveness to incoming inputs (Oliet et al. 2001). To test whether synaptic transmission onto newborn neurons is also modulated in this fashion, the effect of dihydrokainate (DHK, 300 µM), a specific blocker of the astrocytic glutamate transporter GLT-1 (Oliet et al. 2001; Zhang et al. 2009), was tested on evoked postsynaptic responses. We recorded EPSCs from retrovirally labeled granule cells, at 30 dpi, in response to paired pulses delivered to the perforant path (Laplagne et al. 2006). DHK elicited a significant reduction in the EPSC amplitude that was accompanied by an increase in paired pulse ratio (PPR, Fig. 4). The enhanced paired pulse ratio indicates that presynaptic release probability has been reduced by DHK. Thus, astrocytic glutamate transporters enhance release probability and, as a consequence, strengthen excitatory synaptic transmission onto newly generated granule cells.

These results show that astrocytic perisynaptic processes formed on the synapses of newborn neurons are functional and play a role in the synaptic activity of new neurons.

### Structural role of perisynaptic processes on adult-born neurons

**Fig. 5:**
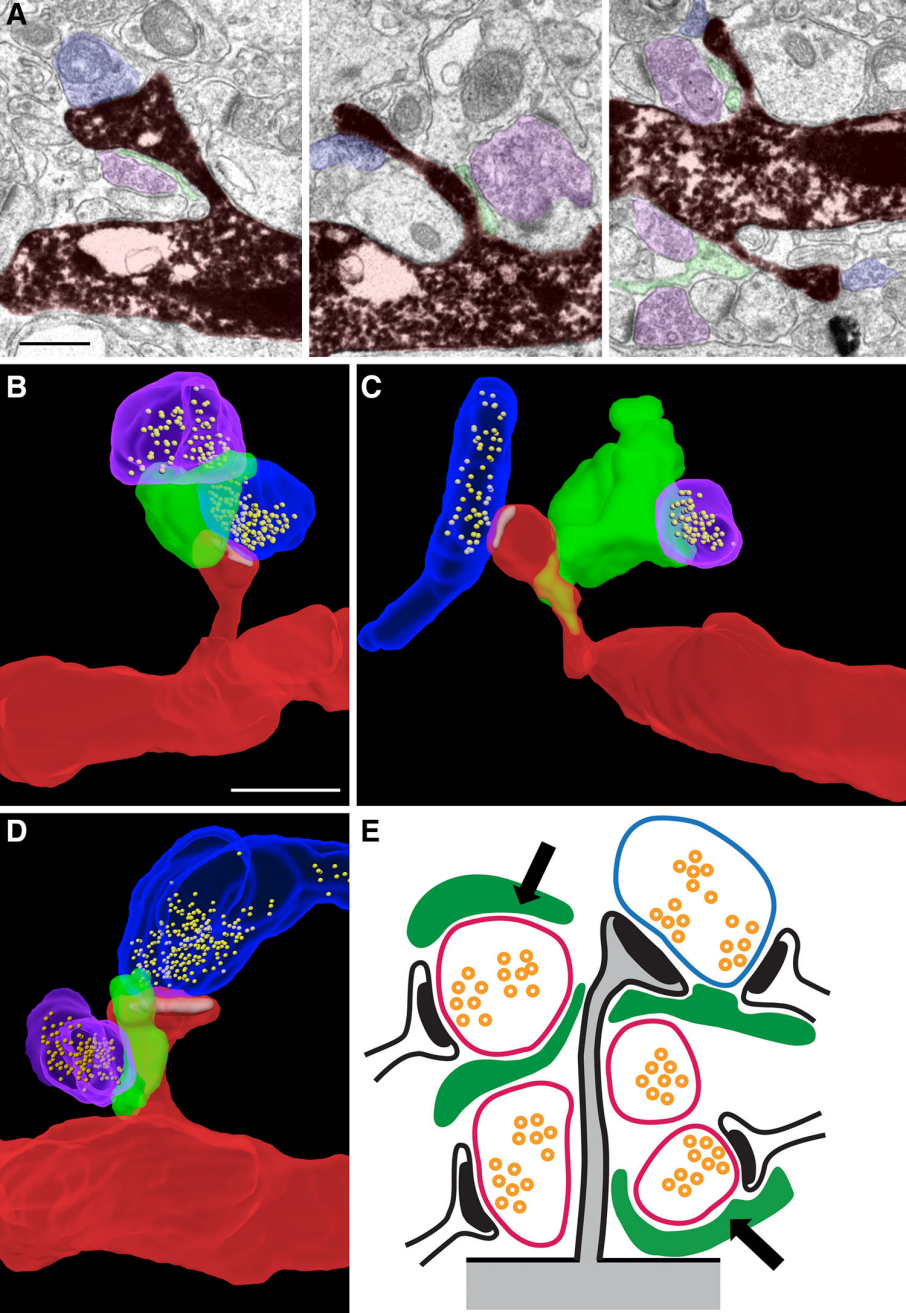
Distribution of perisynaptic processes and of potential presynaptic partners. **a** Electron micrographs of dendritic spines from new neurons (30 dpi, false-colored in *red*) synapsing with an axon terminal (*blue*) and showing an astrocytic process (*green*) intercalated between the spine and a potential presynaptic partner (*pink*). *Scale bar* 0.5 µm. **b**–**d** Three-dimensional reconstructions of dendritic spines (*red*), their presynaptic partner (*blue*) and a potential presynaptic partner (*pink*), separated by an astrocytic process (*green*). For illustrative purpose, only one potential presynaptic partner and the portion of the astrocytic process intercalating between this terminal and the spine has been rendered. *Scale bar* 0.5 µm **e** Schematic representation of the average number of presynaptic terminals and astrocytic processes surrounding a dendritic spine from a new neuron (*gray*), showing four potential presynaptic partners (*pink*) and one synapsing axon terminal (*blue*). Perisynaptic processes (*green*) are shown, two of which intercalate between the spine and two potential presynaptic partners (*arrows*)

Dendritic spines rarely have a straight neck and do not necessarily synapse with the nearest axonal bouton. Since new neurons preferentially contact pre-existing axonal boutons (Toni et al. 2007, 2008), we hypothesized that astrocytic perisynaptic processes, by ensheathing pre-existing axons, may play a structural role in the connectivity of the spines of the newborn neurons. To assess this possibility, we examined the spatial distribution of astrocytic processes and axonal boutons in the vicinity of new dendritic spines. We used serial section electron microscopy and analyzed the volume within a radius of 1 μm around dendritic spines from new neurons at 30 dpi (*n* = 62 spines analyzed). We found that the distance between dendritic spines and the closest astrocytic process ranged between 0 and 100 nm and 81 % of the dendritic spines were touched by an astrocytic process, either on the head or on the neck (Fig. 5). On average, 5.4 axonal boutons were found in a radius of 1 μm from a given spine and could, in principle, make a synapse with it, thereby defining a connectivity fraction of 0.18, similar to the connectivity fraction reported for the CA1 area (Mishchenko et al. 2010) (boutons isolated from the spine by a dendrite or an axon, were not taken into account). For 34 spines for which the volume was fully reconstructed, the potential presynaptic partners not synapsing with the spine represented a total of 150 boutons. Of these, 109 were synapsing already with an unlabeled spine, indicating they were glutamatergic and functional and 41 were not completely comprised in the reconstructed volume. Three potential presynaptic partners were found to synapse with a dendrite, suggesting they were GABAergic and were removed from the analysis. More importantly, for 56 of 147 boutons (38 %, *p* = 0.005 for equal proportions H_0_), an astrocytic process was found intercalated between the boutons and the new spine (Fig. 5). Together, these results show that pre-existing perisynaptic processes intercalate between the new spines and some of their potential presynaptic partners and by doing so, may play a structural role in their connectivity by blocking the access of the spines to some of their potential partners.

## Discussion

In the present study, we examined the formation and function of astrocytic perisynaptic processes on synapses made by adult-born hippocampal neurons. We found that mature perisynaptic processes are present on both afferent synapses (dendritic spines in the dentate gyrus) and efferent synapses (MFT in the CA3 area) of newly formed neurons, regardless of neuronal age or synapse morphology. Perisynaptic processes were mostly recruited from pre-existing astrocytes and shared between newly-formed and pre-existing synapses. In some cases, perisynaptic processes intercalated between spines from new neurons and potential presynaptic partners, suggesting that they might play a structural role in the connectivity of the nascent spines. Finally, the blockade of astrocytic glutamate transporters by DHK indicated that astrocytic glutamate reuptake regulated the synaptic activity of adult-born neurons. Together, these results indicate that astrocytes play a functional and structural role in the integration of adult-born neurons into the hippocampal network by quickly ensheathing their synapses and enhancing their synaptic transmission.

### Perisynaptic processes on newborn neurons

The astrocytic ensheathement of synapses varies considerably between brain areas, from the cerebellum, where nearly all climbing fiber synapses are completely ensheathed by Bergmann glia, to the neocortex, where only 29 % of synapses are partially contacted by astrocytes (Spacek 1985). Here, we found that about 85 % of spine synapses from adult–born neurons are partially ensheathed by a perisynaptic astrocytic process. This proportion is comparable to control granule neurons and to pyramidal neurons of the adult rat CA1 area of the hippocampus, showing a partial ensheathement of about 57–62 % of the Schaffer collateral synapses (Ventura and Harris 1999; Witcher et al. 2007). On the presynaptic side, virtually all of MFT displayed large perisynaptic processes covering a great portion of the surface of the terminals, without reaching the synaptic cleft, consistent with other reports in the CA3 (Rollenhagen et al. 2007). Thus, the perisynaptic processes formed on adult-born neurons exhibited morphological properties typical for granule neurons. It is striking that perisynaptic processes were already present at 14 dpi, when the first dendritic spines and axon terminals of adult-born neurons appear and, surprisingly, astrocytic coverage did not increase further with neuronal maturation. This observation indicates that synaptogenesis is rapidly accompanied by the formation of perisynaptic ensheathement, suggesting a role for perisynaptic glia in the early phase of synapse maturation.

Astrocytic synaptic ensheathement plays a functional role by clearing glutamate released by synaptic activity, with the use of high-affinity glutamate transporters (Bergles and Jahr 1998). Thus, astrocytes contribute to neuronal signaling by modulating excitatory synaptic transmission. Particularly, in the supraoptic nucleus, modulation of glutamatergic transmission was revealed by blocking the astrocytic glutamate transporter GLT1 by the specific GLT1 blocker DHK (Oliet et al. 2001). GLT1 blockade elicited the activation of presynaptic metabotropic glutamate receptors that decreased release probability and, consequently, synaptic efficacy at excitatory synapses was reduced. In adult-born neurons, DHK induced a marked drop in the EPSC amplitude accompanied by a slight increase in paired pulse ratio, also pointing to a presynaptic reduction of glutamate release. Although the subcellular distribution of GLT1 transporters on newly formed perisynaptic processes was not assessed here, the presence of GLT1 transporters on hippocampal astrocytes (Reye et al. 2002) and of presynaptic metabotropic receptors in perforant path synapses of the molecular layer (Maki et al. 1994; Dietrich et al. 1997; Macek et al. 1996) suggests that the effect of DHK on adult-born neurons may be similar to the supraoptic nucleus. Further experiments may, however, clarify the mechanism by which glutamate clearance from the synaptic cleft increased presynaptic release probability on adult-born neurons.

### Plasticity of astrocytic processes

**Fig. 6:**
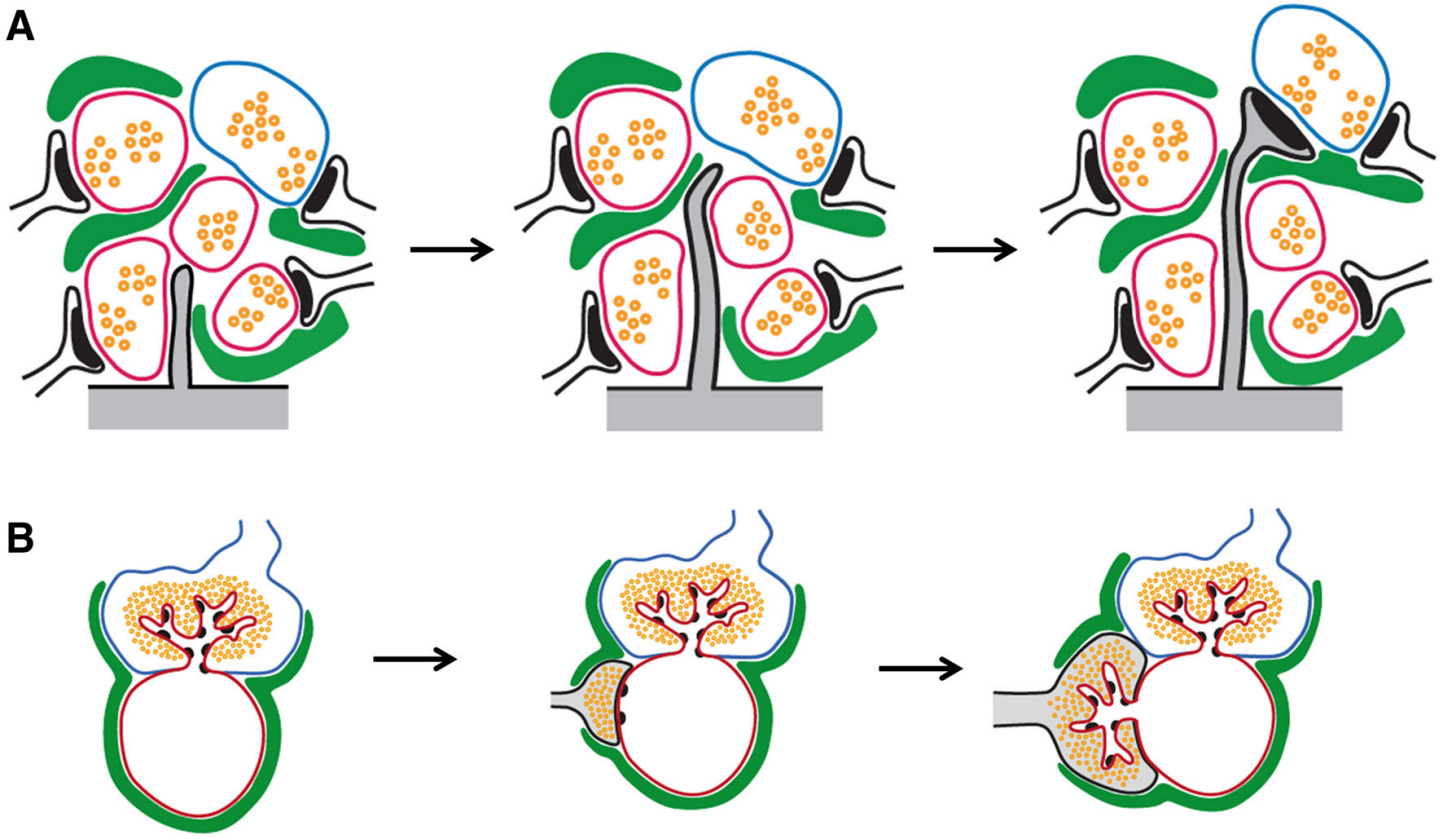
Schematic illustration of the formation of astrocytic perisynaptic processes on spines or MFT from adult–born neurons. **a** Dendritic spines: as a spine from a new neuron extends from its dendrite (*gray*), it touches several potential presynaptic partners (*pink, left panel*). Upon reaching its target axon terminal (*blue*, *middle panel*), the spine forms a multiple-synapse bouton. The perisynaptic process that was present on the pre-existing synapse modifies its structure to ensheathe the newly formed spine (*green*, *right panel*). **b** MFT: as a MFT from a newborn neuron (*gray*) extends, it generally contacts a dendrite (*red*) already synapsing with one or several other MFT (*blue*). The perisynaptic process (*green*) that was present on the pre-existing synapse modifies its structure to ensheathe the newly formed MFT

The relatively small number of astrocytes generated during adulthood suggests that adult-born neurons are principally contacted by pre-existing astrocytes. We, however, do not exclude that some perisynaptic processes may be formed by astrocytes generated during adulthood, prior to the observed newborn neurons. Furthermore, the observation of perisynaptic processes on MSB indicates that pre-existing perisynaptic processes modify their structure to ensheath nascent spines from adult-born neurons, as schematized in Fig. 6. Thus, these observations suggest that pre-existing perisynaptic processes are highly plastic and modulate their structure to establish contact with the nascent spines or boutons of adult-born neurons. These results are consistent with the notion that astrocytes remain plastic throughout adulthood. Indeed, previous reports have shown that astrocytic processes are very plastic and their motility is coordinated with the motility of dendritic spines (Nishida and Okabe 2007; Haber et al. 2006; Hirrlinger et al. 2004; Witcher et al. 2007). Astrocytes extend and retract processes towards nascent dendritic spines in the course of minutes and, interestingly, glial processes are more stable on larger spines, suggesting that they may contribute to spine maturation (Haber et al. 2006). Indeed, when analyzing the motility of astrocytic processes and of their adjacent dendritic spines, Nishida et al. found that astrocytic contact enhanced the lifetime and maturation of nascent dendritic spines (Nishida and Okabe 2007). Also, astrocytes are able to increase their ensheathement of excitatory synapses on dendritic spines during a period of modified neuronal activity in the adult mouse, indicating that their structural plasticity is regulated by neuronal activity (Hirrlinger et al. 2004; Genoud et al. 2006). Further experiments using live-imaging approaches on adult-born neurons and astrocytic processes may determine whether the growth of astrocytic processes precedes or follows the growth and stabilization of dendritic spines of adult-born neurons and to what extent these contacts are necessary for the stable integration of new neurons. However, in view of these studies, we predict that the structural plasticity of astrocytes is necessary to enable the proper integration of adult-born neurons in the hippocampal network and a dysregulation of pre-existing glia may impair the synaptic integration of new neurons in the adult hippocampus. Consistently, the expression of a mutated form of GFAP in the astroglial compartment reduced adult neurogenesis and impaired the development of adult-born neurons in a mouse model of Alexander’s disease (Hagemann et al. 2013).

### Perisynaptic processes and the connectivity of new neurons

Since adult-born neurons preferentially synapse with pre-existing presynaptic partners (Toni et al. 2008, 2007), the specificity of their connectivity can be achieved by the extension of their dendritic spines towards specific targets or by the selective elimination of aberrant connections, or both. When extending in the neuropile, dendritic spines have on average 5.4 axon terminals within their reach, but do not necessarily synapse with the closest axonal bouton (Fig. 6). The choice of presynaptic partner may depend on extrinsic cues: nascent dendritic spines respond to extracellular glutamate by extending towards the source of glutamate (Portera-Cailliau et al. 2003), a mechanism that may result in the formation of MSBs upon glutamate leakage from active synaptic clefts. We can speculate that astrocytes, by clearing glutamate from the synaptic cleft and reducing its diffusion in the extrasynaptic space (Bergles and Jahr 1998), may favor the growth of nascent spines towards the least ensheathed axonal bouton. In this perspective, subcellular changes in GLT1 expression level on perisynaptic processes ensheathing nascent or newly formed synapses may regulate filopodia behavior or the stability of newly formed synapses, questions that may be assessed using immuno-electron microscopy. In addition, by physically obstructing the contact with potential synaptic partners, astrocytes may hinder the formation of contacts with the most ensheathed boutons or guide nascent spines towards a specific presynaptic partner, as has recently been shown during the development of *C. elegans* connectivity (Colon-Ramos et al. 2007). Finally, by secreting molecules that induce synaptogenesis or synaptic plasticity, such as Thrombospondins (Christopherson et al. 2005), d-serine (Panatier et al. 2006), ATP (Pascual et al. 2005), brain-derived neurotrophic factor (Bergami et al. 2008), Glypicans (Allen et al. 2012) or cholesterol (Mauch et al. 2001), astrocytes could induce synaptogenesis in specific territories or on selective partners. In support of this latter idea, glutamate released by astrocytes has been shown to regulate migrating neuroblasts in the rostro-migratory stream (Platel et al. 2010). Our dataset is consistent with all 3 possibilities: Dendritic spines from new neurons were found no further than 100 nm from an astrocytic process, a distance small enough to enable the diffusion of small molecules secreted by these processes towards the nascent protrusion (Witcher et al. 2007). Inversely, glutamate buffering by these processes may influence filopodia behavior. Finally, the ensheathement of surrounding, potential presynaptic partners may limit their accessibility to nascent spines. Thus, with their highly ramified network of processes, astrocytes are ideally positioned to guide the trajectory of the nascent dendritic spines or axon terminals and influence the choice of synaptic partner, a hypothesis schematized in Fig. 6. Consistently, epilepsy reduces perisynaptic glia (Witcher et al. 2010) and also induces the aberrant synaptic integration of newborn neurons (Jessberger et al. 2007). Further experiments aimed at modulating gliotransmission, the structural plasticity of astrocytic processes or the expression of GLT1 transporters and monitoring synaptogenesis with live-imaging microscopy, may determine the role of astrocytes in the synaptic integration of new neurons.

Through their multifaceted function, astrocytes are involved in several steps of adult neurogenesis, from stem cell proliferation to the migration of new neurons. The results presented here indicate that hippocampal astrocytes also contribute to the synaptic integration of new neurons by providing appropriate perisynaptic ensheathement and coupling the astroglial network with adult-born neurons by strengthening the synaptic transmission on these cells. These results are relevant to the role of astrocytes in brain plasticity, transplantation procedures (Han et al. 2013) and in neurodegenerative diseases.

*Authors’ contribution* N.T., A.F.S., and M.K Conceived and designed the experiments. M.K., S.G.T., L.A.M., J.A., V.S., M.V., L.V., J.K.B., M.B. and, K.K.C. performed the experiments. M.K., N.T., S.G.T., L.A.M., J.A., V.S., M.B.,and A.F.A. analyzed the data. M.K., N.T., A.F.S., M.B., and F.H.G.wrote the manuscript. N.T., A.F.S., F.H.G. M.B., and L.V. provided financial support.

## Electronic supplementary material

Below is the link to the electronic supplementary material.

Supplementary Fig. 1 Schematic illustration of the dendritic extension measurements. Dendritic extension = a/b × 100; a is the distance between the center of the cell body and the tip of the longest dendrite; b is the distance between the center of the cell body and the end of the molecular layer. Supplementary material 1 (JPEG 952 kb)

Supplementary Fig. 2 GFP expression in GFAP-GFP and Aldh1l1-GFP mice. **A.** Confocal micrograph (maximal intensity projection) of the dentate gyrus of a GFAP-GFP (left) and an Aldh1l1-GFP (right) mouse. Scale bar: 40 µm. **B.** Proportion of GFAP-immunostained cells that also express GFP in both mouse models (Student’s t-test, *** : p < 0.001). Supplementary material 2 (JPEG 2255 kb)

Supplementary Fig. 3 MFT area increases with the age of adult-born neurons. Histogram showing the increase of MFT area with adult-born neuron maturation (One-way Anova, F(4,32) = 6, p < 0.001, n = 5 to 12 MFT per timepoint; 14, 17dpi < 28,56 dpi) *: p < 0.05. Supplementary material 3 (JPEG 761 kb)
