# Supplementary figures and images for: Pre-existing astrocytes form functional perisynaptic processes on neurons generated in the adult hippocampus
Source: Brain Struct Funct. 2014 Apr 19;220(4):2027–42. doi: 10.1007/s00429-014-0768-y (PMC4481333; doi:10.1007/s00429-014-0768-y)

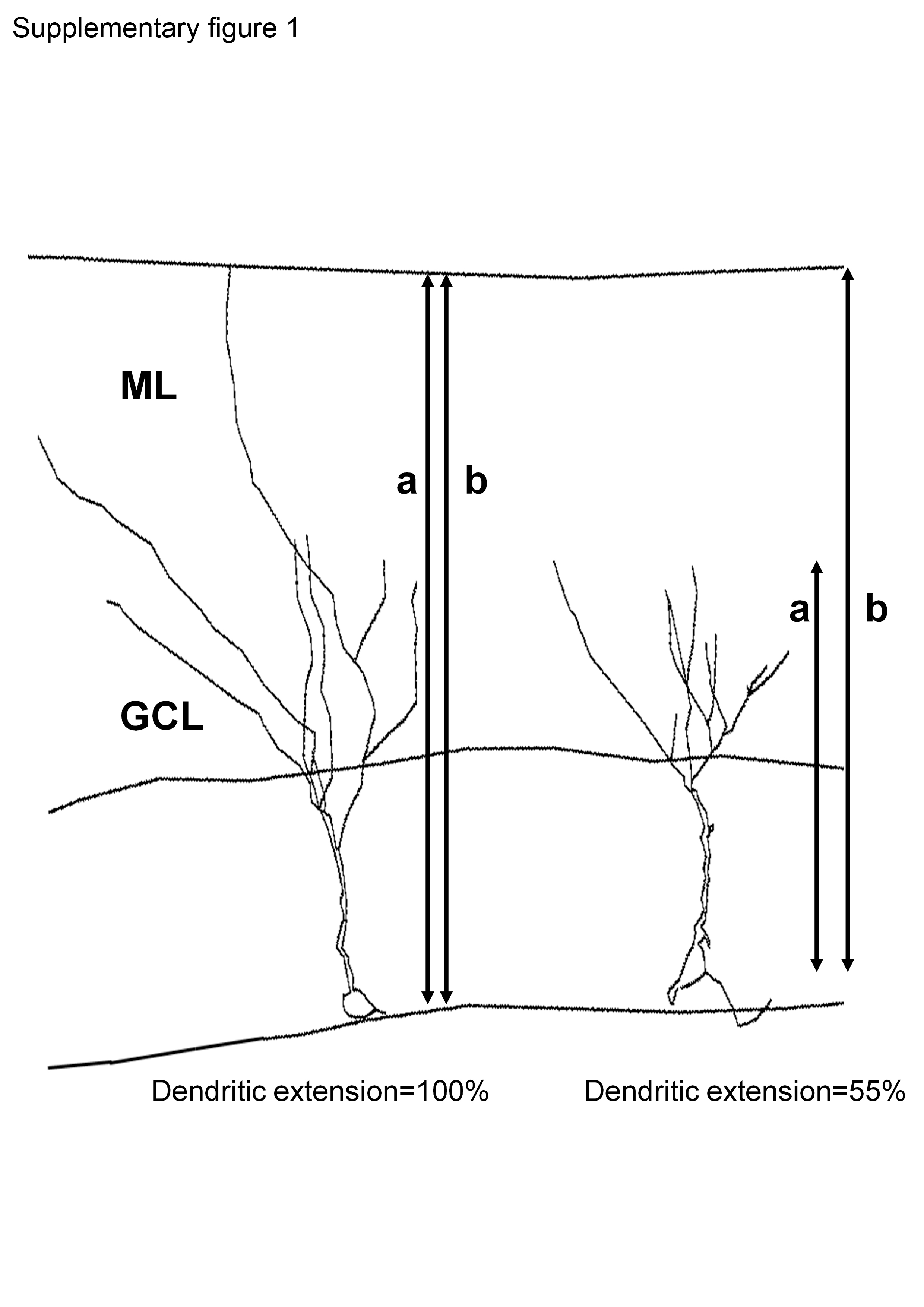

Supplement: Supplementary file 1 — Schematic illustration of the dendritic extension measurements. Dendritic extension = a/b × 100; a is the distance between the center of the cell body and the tip of the longest dendrite; b is the distance between the center of the cell body and the end of the molecular layer. Supplementary material 1 (JPEG 952 kb) [file 429_2014_768_MOESM1_ESM.jpg]

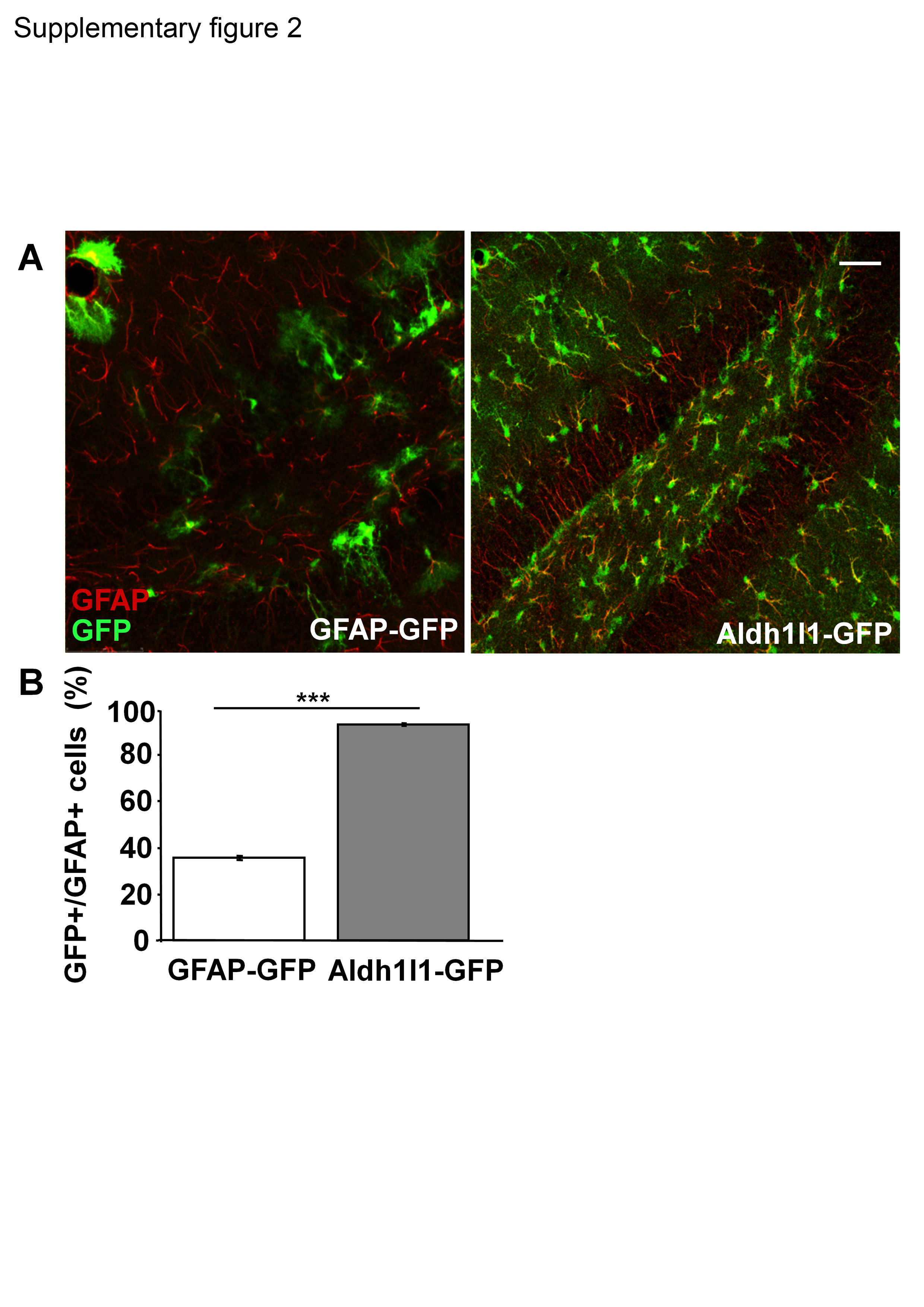

Supplement: Supplementary file 2 — GFP expression in GFAP-GFP and Aldh1l1-GFP mice. A. Confocal micrograph (maximal intensity projection) of the dentate gyrus of a GFAP-GFP (left) and an Aldh1l1-GFP (right) mouse. Scale bar: 40 µm. B. Proportion of GFAP-immunostained cells that also express GFP in both mouse models (Student’s t-test, *** : p < 0.001). Supplementary material 2 (JPEG 2255 kb) [file 429_2014_768_MOESM2_ESM.jpg]

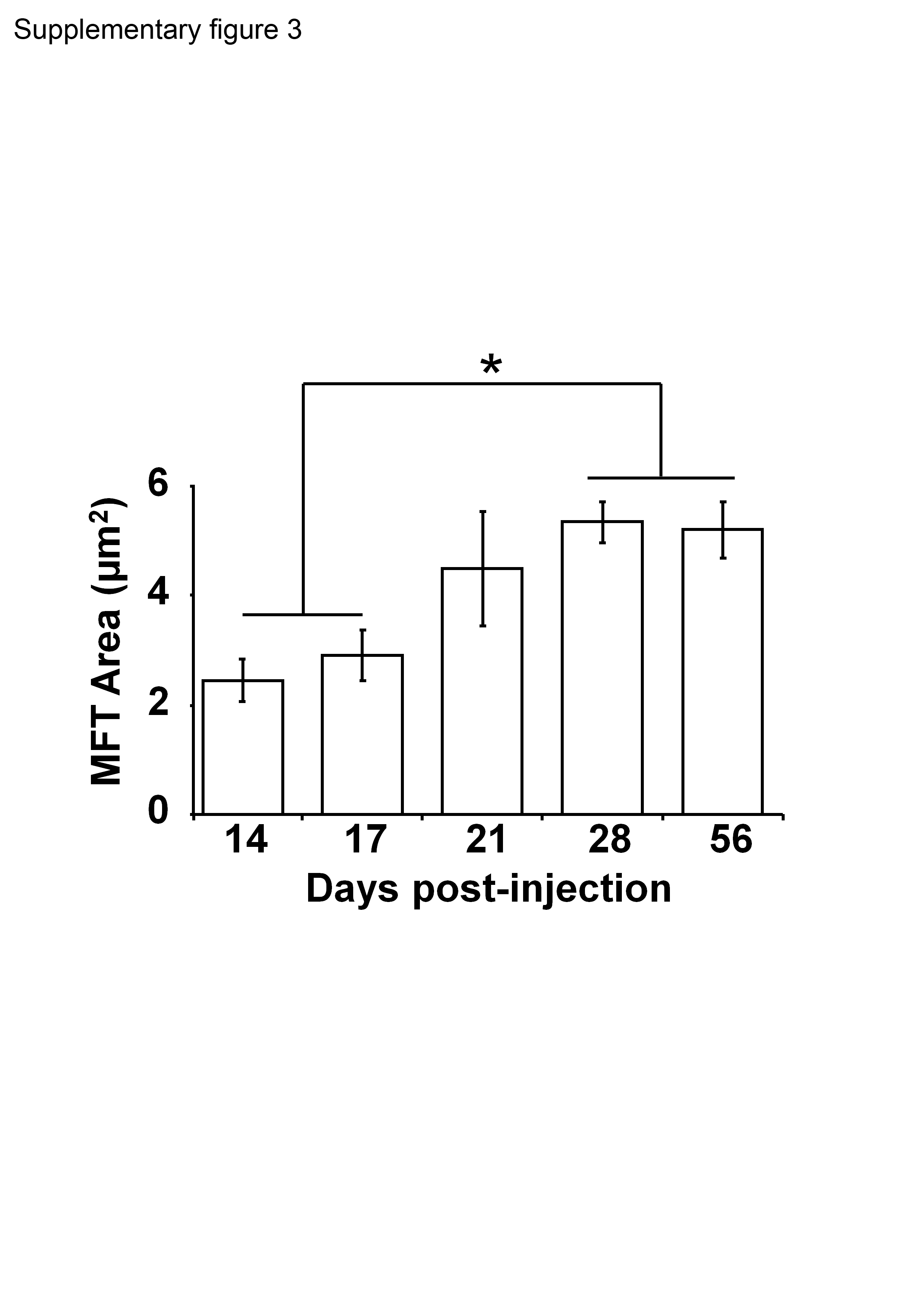

Supplement: Supplementary file 3 — MFT area increases with the age of adult-born neurons. Histogram showing the increase of MFT area with adult-born neuron maturation (One-way Anova, F(4,32) = 6, p < 0.001, n = 5 to 12 MFT per timepoint; 14, 17dpi < 28,56 dpi) *: p < 0.05. Supplementary material 3 (JPEG 761 kb) [file 429_2014_768_MOESM3_ESM.jpg]
